# Supplementary material for: Developing and testing the feasibility of a new internet-based intervention–A case study of people with stroke and occupational therapists
Source: PLoS One. 2023 Dec 28;18(12):e0296364. doi: 10.1371/journal.pone.0296364 (PMC10754454; doi:10.1371/journal.pone.0296364)
Supplement: Attachment — Submitted filename: Review 08.07.2022 PONE.docx [file pone.0296364.s001.docx]

# Review 08.07.2022 P-ONE.

Thank you for having the opportunity to review the manuscript **Developing and testing the feasibility of a new internet-based intervention– a case.**

Overall

The paper presents an interesting research study aiming to enhance knowledge on developing stroke rehabilitation within the research field of occupational therapy. However, there are some concerns regarding the presentation of the study.

The title embraces the content of the study. The abstract includes relevant information, and the key words are not already used in the title.

The introduction explains the background of the study grounded in updated literature and the rationale of the study states the significance for occupational therapy.

Page 3

- lines 69 -70 – please insert a well-known definition on occupational balance and one or more references.
- Lines 75- 76 – please rephrase the sentence and include a well-known definition of self-management.
- Line 77 – please clarify the term “facility” in this context.
- Line 78 – please give examples of common unfulfilled needs
- Line 79 – please develop the argument for a new mode of service. Why will a more stable delivery of existing rehabilitation services not be sufficient?

Page 4

- Line 89 – please define the concept of empowerment and provide relevant references

The methods and materials used are overall well presented and argued. Considerations are taken to provide appropriate and trustworthy procedures and ethical considerations are accounted for.

I recommend further clarity of the inclusion criteria:

- moderate disability or good recovery
- motivated to change their activities in everyday life

And an elaboration on how the screening for inclusion was undertaken as the inclusion criteria seem quite unclear.

Moreover, please develop the argumentation for data collection tools related to the data collection periods – and associate the data collection to the concept of empowerment as well as activities of daily life and management strategies.

In this section the authors may also want to state and reflect on their preunderstanding.

The result section present findings relevant to the aim of the study. The table adequately enhance the presentation of the material.

In table 3 delivery is mentioned twice. How come?

Page13

- line 271 – it is unclear who provide the narrations

Page 15

- lines 330 - erase small line

The discussion section considers the findings in view of previously reported research and avenues for future research is suggested.

Page 21

- please elaborate on the feasibility of SEE in the clinical setting of the OT – how time consuming is the introduction to and the use of the SEE compared to usual practice? How did the change in the OT practice affect the practice of other professionals in the interdisciplinary teams?

The tentative reflections on the findings may be in want of more nuances and critical reflections. How does these findings differ from or develop already well-known research? Please develop and elaborate on the statements concerning the relevance for occupational therapy practice as the findings are based on very few persons and apparently one single rehabilitation setting – e.g., please expand the discussion on, why variation did not enter into the inclusion as this is an issue in relation to the quality of the research, and how these findings relate to the concept of empowerment and occupational therapy theories in the context of stroke rehabilitation and occupational therapy.

Reflection on need for future research and the methodological considerations present very important reflections that may well have been elaborated, included in, and qualified the discussion.
