# Supplementary material for: Developing and testing the feasibility of a new internet-based intervention–A case study of people with stroke and occupational therapists
Source: PLoS One. 2023 Dec 28;18(12):e0296364. doi: 10.1371/journal.pone.0296364 (PMC10754454; doi:10.1371/journal.pone.0296364)
Supplement: Attachment — Submitted filename: Response to Reviewers 220824.docx [file pone.0296364.s002.docx]

**Response to Editor and Reviewer**

**Journal Requirements:**

When submitting your revision, we need you to address these additional requirements.

1. Please ensure that your manuscript meets PLOS ONE's style requirements, including those for file naming. The PLOS ONE style templates can be found at 
[https://journals.plos.org/plosone/s/file?id=wjVg/PLOSOne_formatting_sample_main_body.pdf](https://eur02.safelinks.protection.outlook.com/?url=https%3A%2F%2Fjournals.plos.org%2Fplosone%2Fs%2Ffile%3Fid%3DwjVg%2FPLOSOne_formatting_sample_main_body.pdf&data=05%7C01%7C%7C5cf99fb7c64a440c214208da6e4aea47%7C5453408ba6cd4c1e8b1018b500fb544e%7C1%7C0%7C637943565270709488%7CUnknown%7CTWFpbGZsb3d8eyJWIjoiMC4wLjAwMDAiLCJQIjoiV2luMzIiLCJBTiI6Ik1haWwiLCJXVCI6Mn0%3D%7C3000%7C%7C%7C&sdata=JZw8ypvCAulQUkjqvJuBHxbGE%2Fqoi2fZ7lRG6zMLQbA%3D&reserved=0) and 
[https://journals.plos.org/plosone/s/file?id=ba62/PLOSOne_formatting_sample_title_authors_affiliations.pdf](https://eur02.safelinks.protection.outlook.com/?url=https%3A%2F%2Fjournals.plos.org%2Fplosone%2Fs%2Ffile%3Fid%3Dba62%2FPLOSOne_formatting_sample_title_authors_affiliations.pdf&data=05%7C01%7C%7C5cf99fb7c64a440c214208da6e4aea47%7C5453408ba6cd4c1e8b1018b500fb544e%7C1%7C0%7C637943565270709488%7CUnknown%7CTWFpbGZsb3d8eyJWIjoiMC4wLjAwMDAiLCJQIjoiV2luMzIiLCJBTiI6Ik1haWwiLCJXVCI6Mn0%3D%7C3000%7C%7C%7C&sdata=wrktT9do3NYUHvHNG6F0msjJASpB3LYn6odU9%2BdzPPo%3D&reserved=0)

***Reply:*** Thank you for your reminder. We have carefully reviewed the manuscript against PLOS ONE style requirements and made subsequent changes.

2. Please provide additional details regarding participant consent. In the ethics statement in the Methods and online submission information, please ensure that you have specified what type you obtained (for instance, written or verbal, and if verbal, how it was documented and witnessed). If your study included minors, state whether you obtained consent from parents or guardians. If the need for consent was waived by the ethics committee, please include this information.

***Reply:*** Thank you for your comment. We have clarified how potential participants were informed throughout the inclusion process and how oral and written informed consent was achieved by all participants. (Method section, recruitment)

3. Thank you for stating the following financial disclosure:

This study was supported by grants from the Kamprad Family Foundation for Entrepreneurship, Research & Charity. The funder had no role in the study.

Please state what role the funders took in the study.  If the funders had no role, please state: "The funders had no role in study design, data collection and analysis, decision to publish, or preparation of the manuscript." 
If this statement is not correct you must amend it as needed. 
Please include this amended Role of Funder statement in your cover letter; we will change the online submission form on your behalf.

***Reply:*** We agree to the suggested statement and have added the sentence of the role of the funder in the cover letter.

4. In your Data Availability statement, you have not specified where the minimal data set underlying the results described in your manuscript can be found. PLOS defines a study's minimal data set as the underlying data used to reach the conclusions drawn in the manuscript and any additional data required to replicate the reported study findings in their entirety. All PLOS journals require that the minimal data set be made fully available. For more information about our data policy, please see [http://journals.plos.org/plosone/s/data-availability](https://eur02.safelinks.protection.outlook.com/?url=http%3A%2F%2Fjournals.plos.org%2Fplosone%2Fs%2Fdata-availability&data=05%7C01%7C%7C5cf99fb7c64a440c214208da6e4aea47%7C5453408ba6cd4c1e8b1018b500fb544e%7C1%7C0%7C637943565270709488%7CUnknown%7CTWFpbGZsb3d8eyJWIjoiMC4wLjAwMDAiLCJQIjoiV2luMzIiLCJBTiI6Ik1haWwiLCJXVCI6Mn0%3D%7C3000%7C%7C%7C&sdata=44w%2BVvkMlm1HrXVLsDgkr4duB3dW%2F0HAqxsWer%2Fl%2FOw%3D&reserved=0).

Upon re-submitting your revised manuscript, please upload your study’s minimal underlying data set as either Supporting Information files or to a stable, public repository and include the relevant URLs, DOIs, or accession numbers within your revised cover letter. For a list of acceptable repositories, please see <http://journals.plos.org/plosone/s/data-availability#loc-recommended-repositories>. Any potentially identifying patient information must be fully anonymized.

Important: If there are ethical or legal restrictions to sharing your data publicly, please explain these restrictions in detail. Please see our guidelines for more information on what we consider unacceptable restrictions to publicly sharing data: <http://journals.plos.org/plosone/s/data-availability#loc-unacceptable-data-access-restrictions>. Note that it is not acceptable for the authors to be the sole named individuals responsible for ensuring data access.

We will update your Data Availability statement to reflect the information you provide in your cover letter.

***Reply:*** The Data Availability statement have been updated and are provided in our cover letter. *Unfortunately, we cannot share our data openly due to Swedish regulations. However, more clear information is provided on where to ask for permission to get access the archived data at our university.*

5. Your ethics statement should only appear in the Methods section of your manuscript. If your ethics statement is written in any section besides the Methods, please delete it from any other section.

***Reply:*** Thank you for your reminder. Our ethical statement can only be found under the method section.

6. Please include a copy of Table 4 which you refer to in your text on page 12.

***Reply:*** Thank you for bringing this to our attention. There must have been a mistake, Table 4 should of course be included in the manuscript, which it is now.

**Additional Editor Comments:**

- It is appreciated that some limitations of the study are discussed, if possible please include a comment about the small sample size and the limited age range of the participants.

***Reply:*** Thank you for your comment. The small sample size and the limited age range of the participants is now discussed in the methodological considerations. Also, the limitation of the case study design is more clearly outlined in the whole discussion.

**Comments from reviewer 1:**

Reviewer #1: Thank you for having the opportunity to review the manuscript “Developing and testing the feasibility of a new internet-based intervention– a case”

Overall
The paper presents an interesting research study aiming to enhance knowledge on developing stroke rehabilitation within the research field of occupational therapy. However, there are some concerns regarding the presentation of the study.
The title embraces the content of the study. The abstract includes relevant information, and the key words are not already used in the title.
The introduction explains the background of the study grounded in updated literature and the rationale of the study states the significance for occupational therapy.

***Reply:*** Thank you for the overall positive response!

Page 3
- lines 69 -70 – please insert a well-known definition on occupational balance and one or more references.
- Lines 75- 76 – please rephrase the sentence and include a well-known definition of self-management.
- Line 77 – please clarify the term “facility” in this context.
- Line 78 – please give examples of common unfulfilled needs
- Line 79 – please develop the argument for a new mode of service. Why will a more stable delivery of existing rehabilitation services not be sufficient?

Page 4
- Line 89 – please define the concept of empowerment and provide relevant references

***Reply:*** Thank you for your comments on the introduction that all strengthens it even further. We have clarified the concepts an added some new references. The unclear sentences has been clarified and arguments for internet-based services has been developed. We have chosen not to define the concept of empowerment per se, as we use empower in relation to the (definition of the) concept of self-management. We also more clearly refer, below the heading intervention, where more information about the knowledge base/ theoretical base of SEE can be found.

The methods and materials used are overall well presented and argued. Considerations are taken to provide appropriate and trustworthy procedures and ethical considerations are accounted for.

I recommend further clarity of the inclusion criteria:
- moderate disability or good recovery
- motivated to change their activities in everyday life

And an elaboration on how the screening for inclusion was undertaken as the inclusion criteria seem quite unclear.

Moreover, please develop the argumentation for data collection tools related to the data collection periods – and associate the data collection to the concept of empowerment as well as activities of daily life and management strategies.

***Reply:*** Thank you for your comments. The inclusion criteria have been clarified as well as the steps of the inclusion process. The choice of data collection methods has been further explained in relation activities of daily life and management strategies. The data collection periods has been clarified. As self-management is included in the theoretical foundation of SEE (and not empowerment per se), we measure self-efficacy that is a important in relation to both self-management and empowerment.

In this section the authors may also want to state and reflect on their preunderstanding.

***Reply:*** We have chosen to make changes in the methodological considerations (Discussion section) by developing the sentences about our pre-understanding and how it was handled to remain open during analysis.

The result section present findings relevant to the aim of the study. The table adequately enhance the presentation of the material.
In table 3 delivery is mentioned twice. How come?

Page13
- line 271 – it is unclear who provide the narrations
Page 15
- lines 330 - erase small line

***Reply:*** Thank you, the use of delivery twice in Table 3 was a mistake which we now have corrected. The other small errors have also been corrected.

The discussion section considers the findings in view of previously reported research and avenues for future research is suggested.
Page 21
- please elaborate on the feasibility of SEE in the clinical setting of the OT – how time consuming is the introduction to, and the use of the SEE compared to usual practice? How did the change in the OT practice affect the practice of other professionals in the interdisciplinary teams?

***Reply:*** Thank you for your comments. This study was the first test of the SEE, which point to a need of development of the routines of the delivery intervention itself as well as the education of professionals. We therefore now suggest in the manuscript that these questions can be better answered in our forthcoming feasibility studies with amendments in place.

The tentative reflections on the findings may be in want of more nuances and critical reflections. How does these findings differ from or develop already well-known research? Please develop and elaborate on the statements concerning the relevance for occupational therapy practice as the findings are based on very few persons and apparently one single rehabilitation setting – e.g., please expand the discussion on, why variation did not enter into the inclusion as this is an issue in relation to the quality of the research, and how these findings relate to the concept of empowerment and occupational therapy theories in the context of stroke rehabilitation and occupational therapy.
Reflection on need for future research and the methodological considerations present very important reflections that may well have been elaborated, included in, and qualified the discussion.

***Reply:*** Thank you for your comments which provided the opportunity to further improve our discussion. We agree with the reviewer and have made clarifications requested in both the discussion and methodological considerations to strengthen manuscript. The results are clearer discussed in relation to the limitations of the case study design. Arguments are provided for the study design in relation to MRC guidelines for developing complex interventions and more clearer suggestions for forthcoming research in the feasibility phase is outlined. Also, the paragraph with methodological considerations have been developed to more critical discuss the limitations. The results contribution to research and theory in the area has been outlined in terms of how the results differ or develop existing knowledge.
