# Supplementary material for: Developing and testing the feasibility of a new internet-based intervention–A case study of people with stroke and occupational therapists
Source: PLoS One. 2023 Dec 28;18(12):e0296364. doi: 10.1371/journal.pone.0296364 (PMC10754454; doi:10.1371/journal.pone.0296364)
Supplement: Attachment — Submitted filename: Response to Editor and Reviewer 230517.docx [file pone.0296364.s003.docx]

**Response to Editor and Reviewers**

**Reviewers’ comments to the author**

**Reviewer #1:** The paper presents an interesting research study aiming to enhance knowledge on developing stroke rehabilitation within the research field of occupational therapy. The manuscript is a technically sound piece of scientific research. Methods have been conducted and reported rigorously. Discussions and conclusion are appropriately based on the results presented. All previous comments has been addressed. Hence, this manuscript is now acceptable for publication.

**Replay:** *Thank you!*

**Reviewer #2:**

Abstract
Introduction need to be concrete showing the needs for this study
Method lack of information how the data is collected, who are the participants and number of participants, demo profile of the participants, how the data were analyzed
Findings - poorly written

**Replay:** *Thank you for your comments that help us to improve the abstract. We have now clarified the need for the study, the number of participants, given some demographic data and given examples of data collection methods. The analyse method “pattern matching” is already mentioned in the abstract. We do not understand why the findings is poorly written as it contains the names of categories identified in accordance with how results from qualitative case studies are presented.*

Results
-should include demo profile of the participants, level of education, severity of stroke

The authors should consider quantitative survey instead of using qualitative case study methodology for this study.
The authors should focus perhaps user / patients responds and OT feedback/respond in different study.

**Replay:** *The demographics of the participants is presented in Table 1 in the method section. It includes level of education as well as severity of disability. No other information about level of the stroke severity at the onset was available in the database at the clinic.*

*Our aim is not suitable for a quantitative study. We have clarified why we chose to include both clients and occupational therapist in the same study in accordance with guidelines of qualitative case studies and in relation to the aim of our study. As this was the initial test of SEE, it was favorable to include both groups of users (those who received or delivered SEE) in the case study to provide for a comprehensive understanding of different perspectives See method, section for design. We have also added text in the discussion about the need of a quantitative approach in forthcoming feasibility studies****.***

*In the methodological considerations we already also discuss our choice of design in relation to the aim of the study. Also, in the beginning of the discussion, we already emphasizes that the results should be in considered in the light of the case study design and the fact that this initial clinical testing of SEE was limited to one rehabilitation setting.*

*In addition, this study follows the MRC guidelines for how complex interventions are developed and evaluated and provided a base for the selection of the study design. As described in the introduction lines 87-97, SEE are in development phase of initially testing the prototype in clinical practice. As the intervention is new (both in terms of delivery and content), it is important to identify possible alterations and improvements before a larger-scale feasibility study is performed. Testing SEE on a few cases, closely following both clients and clinical occupational therapists (OTs) during pre- and postintervention, can provide important knowledge of SEE’s (i) feasibility and acceptability, with a focus on delivery and content, as well as (ii) experienced changes and outcomes.*

**Has the statistical analysis been performed appropriately and rigorously?**

Reviewer #2: No

***Reply:*** *This is not a quantitative study. It is a qualitative descriptive case study and no rigorous statical analysis was appropriate.*

**Have the authors made all data underlying the findings in their manuscript fully available?**

Reviewer #2: No

***Reply:*** *Unfortunately, we cannot share our data openly due to Swedish regulations. We have already explained this by giving information on how to ask for permission to get access the data at our university as follows: “The data underlying this study cannot be made publicly available in open databases due to ethical and legal reasons, according to Swedish law and regulations. Further, the ethical approval for the study does not admit that data are openly shared. The ethical application and approval for the study can be requested from the Swedish Ethical Review Authority (Dnr: 2019-04993). Data are archived at Luleå University of Technology (LTU) in accordance with Swedish law and regulations and interested researchers may only obtain data after receiving approval from the Swedish Ethical Review Authority. Requests to access data can be sent to the data protection office at LTU,* [*registrator@ltu.se*](mailto:registrator@ltu.se) *No contact with authors is necessary to request data exchange.”*
