# Supplementary material for: Developing and testing the feasibility of a new internet-based intervention–A case study of people with stroke and occupational therapists
Source: PLoS One. 2023 Dec 28;18(12):e0296364. doi: 10.1371/journal.pone.0296364 (PMC10754454; doi:10.1371/journal.pone.0296364)
Supplement: Attachment — Submitted filename: Response to Editor and Reviewer 231127.docx [file pone.0296364.s004.docx]

**Response to Editor and Reviewers**

**Journal Requirements:**

Please review your reference list to ensure that it is complete and correct. If you have cited papers that have been retracted, please include the rationale for doing so in the manuscript text or remove these references and replace them with relevant current references. Any changes to the reference list should be mentioned in the rebuttal letter that accompanies your revised manuscript. If you need to cite a retracted article, indicate the article’s retracted status in the References list and also include a citation and full reference for the retraction notice.

***Reply:*** *The reference list has been updated with full details. Similarly, three new references (no.15-18) have been added following clarifications in the introduction.*

**Review Comments to the Author:**

Reviewer #3: This paper investigated the PLOS ONE manuscript "Developing and testing the feasibility of a new internet-based intervention– a case study of people with stroke and occupational therapists." I think this paper needs minor revision to improve the rationale and gap in knowledge of the study. Specific comments for each section are below:

***Reply:*** *Thank you for the overall positive response and that only minor revisions remain.*

Introduction
Page 3, Line79: Before going to “A new mode of service”, please give the rationale or gap in knowledge why internet-based intervention could be fulfilled in the future stroke service, it would be helpful to insert addition in the one or two sentences.

***Reply:*** *Thank you for bringing this to our attention, we have now developed the rationale for the study.*

Page 4, Line 91: the MRC guideline needs to give a full name.

Methods
Page 7, Line 153: What is the Swedish 1177? Is it referring to a health care advice phone or hotline? Please clarify this point.

Page 7, Line 163: It would be helpful to provide a brief of web program in which it consists of 8 modules. Please clarify this point that help readers to understand the mechanism of internet-based intervention.

***Reply:*** *Thank you for bringing this to our attention, clarifications have been made as requested.*

Result
Page 12, Line 264: authors mentioned Table 4, where is table 4?
Page 14, Line 303: authors mentioned Table 4, where is table 4?
Page 15, Line 313: authors mentioned Table 4, where is table 4?
Page 17, Line 378: authors mentioned Table 4, where is table 4?

***Reply:*** *Table 4 is already in the manuscript. The Table can be found directly when it is mentioned the first time in the results. It is placed under Table 3 on page 14.*

Page 17, Line 379-381: It was the only Oval-pd and OBQ to support the statement "Having established a new approach to everyday life and experiencing changes". Is there a section for results from POES, WAI, Lisat-11, and GSE-10 to support this? You mentioned four months of follow-up in Table 2 (iv). There is a point in this paper that would strengthen it and make it more understandable to the reader.

***Reply:*** We have clarified that the (results in the) category are based on data both from interviews and assessments. We have also made adjustments in the text and added a sentence about the other assessment not previously mentioned.

Discussion
Page 24, Line 545-549: “Client 2 in category B, who chose not to establish an activity plan and continue the process without guidance from the OT, did not achieve positive changes that were reflected in the assessments. However, the qualitative descriptions (from both client 2 and the OT) reflected that those changes in everyday life had been implemented successfully.” It is likely that these two messages contradict each other. Please clarify this point.

***Reply:*** *The requested clarification has been made.*
